# Perioperative Care of a Child With Miller-Dieker Syndrome

**DOI:** 10.14740/jmc5320

**Published:** 2026-06-03

**Authors:** Samantha A. Currier, Lance M. Relland, Joseph D. Tobias

**Affiliations:** a Department of Anesthesiology, Doctors Hospital and OhioHealth, Columbus, OH, USA; b Department of Anesthesiology and Pain Medicine, Nationwide Children’s Hospital, Columbus, OH 43205, USA; c Department of Anesthesiology and Pain Medicine, The Ohio State University College of Medicine, Columbus, OH, USA

**Keywords:** Miller-Dieker syndrome, Lissencephaly, Seizure, Pediatric anesthesiology

## Abstract

Miller-Dieker syndrome (MDS), also known as Miller-Dieker lissencephaly syndrome or chromosome 17p13.3 deletion syndrome, is a rare chromosomal disorder (microdeletion syndrome) characterized by lissencephaly, altered facial features, and neurologic disability. Classic phenotypic findings include a prominent forehead, micrognathia, and a short nose. Children with MDS are often born with associated end-organ involvement including congenital heart disease as well as motor and neurologic impairments including seizures. Progressive central nervous system involvement frequently leads to upper airway and swallowing dysfunction with recurrent bouts of aspiration, pneumonia, and respiratory failure. We present a 3-year-old child with MDS who presented for anesthetic care for a cystoscopy with ureteroscopy, lithotripsy, and ureteral stent exchange. We explore the genetic history of this disorder, outline end-organ involvement with anesthetic implications, and discuss potential options for perioperative care.

## Introduction

Miller-Dieker syndrome (MDS), also known as Miller-Dieker lissencephaly syndrome or chromosome 17p13.3 deletion syndrome, is a rare chromosomal disorder (microdeletion syndrome) that presents with lissencephaly, altered facial features, and neurologic disability [1–4]. Classic phenotypic findings include a prominent forehead, micrognathia, and a short nose. While this constellation of clinical findings was likely described in postmortem examinations prior to 1963, the disorder was first officially reported as a lissencephaly syndrome by Dr. Miller in 1963 and Dr. Dieker et al in 1969 [5, 6]. Because of their independent descriptions of this disorder in the 1960s, the syndrome was officially termed MDS in recognition of their contributions to its description and identification of this rare genetic condition.

MDS is uncommon, with an incidence of approximately 1 in 100,000 live births. It results from a deletion involving 26 genes within the MDS locus at chromosome 17p13.3, causing monosomy [7]. This specific location on chromosome 17 is a known unstable region of the human genome and is the location of various other mutations resulting in genetic-based neurologic disorders. The majority of MDS cases are due to a sporadic mutation. The contiguous gene mutation involving 17p13.3 results in faulty structural development of the central nervous system (CNS), particularly lissencephaly, a characteristic finding in MDS [3].

Children with MDS are often born with other associated end-organ involvement including congenital heart defects as well as motor and neurologic impairments. Progressive CNS involvement frequently leads to seizures as well as upper airway and swallowing dysfunction with recurrent bouts of aspiration, pneumonia, and respiratory failure. We present a 3-year-old child with MDS who presented for anesthetic care for a cystoscopy with ureteroscopy, lithotripsy, and stent exchange. We explore the genetic history of this disorder, outline end-organ involvement, and discuss potential options for perioperative care.

## Case Report

Review of this case and presentation in this format followed the guidelines of the Institutional Review Board of Nationwide Children’s Hospital. Written consent was obtained for the use of deidentified patient information for publication. This review was conducted in compliance with the ethical standards of the responsible institution on human subjects as well as with the Helsinki Declaration.

The patient was a 3-year-old child (17.3 kg) with MDS who presented for anesthetic care for a cystoscopy with ureteroscopy, lithotripsy, and ureteral stent exchange. Prenatal and birth history included a scheduled cesarean section at 39 weeks of gestation due to concerns for associated congenital anomalies that were identified using prenatal ultrasound and magnetic resonance imaging (MRI). Based on the MRI, which showed lissencephaly, colpocephaly, and dysgenesis of the corpus callosum, a prenatal diagnosis of MDS was suggested by the pediatric genetics consultant. In utero complications included polyhydramnios in the setting of maternal gestational diabetes treated with metformin. Following delivery, the infant required only routine care and stabilization while remaining at the facility to promote bonding time with his mother while she recovered. During a 60-day admission to the neonatal intensive care unit (NICU) at a pediatric specialty hospital, subsequent care for the infant confirmed his diagnosis of MDS via chromosomal analysis (microarray) and included further evaluation by various subspecialty pediatric services such as neurology and otolaryngology. Initial physical examination revealed retro/micrognathia, a high arched palate, wide anterior fontanel, and a sacral dimple. A repeat MRI confirmed the findings noted on the in utero study (Fig. 1). Additional associated congenital anomalies included a large patent foramen ovale, which subsequently resolved without medical or surgical intervention. In addition, he was found to aspirate all consistencies of oral intake during a video swallow study and subsequently underwent gastrostomy tube placement for feeding. The patient was eventually discharged home from this initial admission on room air, but at the time of the preoperative evaluation for the aforementioned procedures, he had a baseline supplemental oxygen requirement of 0.5–1 L/min via nasal cannula during the day and used bilevel positive airway pressure (BiPAP) intermittently at night and during naps (BiPAP settings: inspiratory positive airway pressure (IPAP) 14 cm H_2_O and expiratory positive airway pressure (EPAP) 8 cm H_2_O). In the time between his NICU hospitalization and arrival for the reported procedures, he was diagnosed with the following additional medical conditions: global developmental delay, intractable focal epilepsy, refractory infantile spasms, obstructive sleep apnea, history of respiratory syncytial virus infection, small for gestational age, and kidney stones.

**Figure 1:**
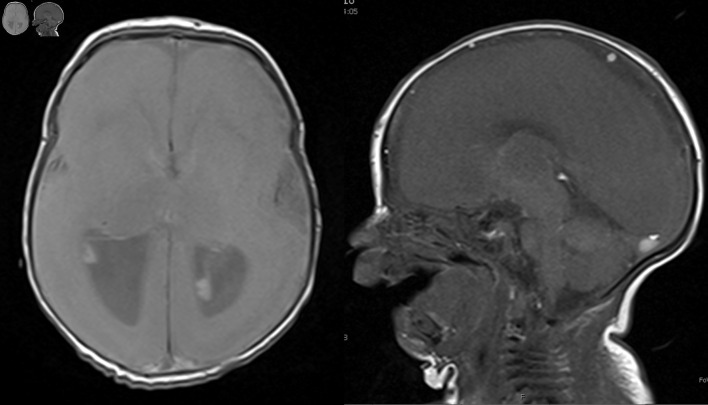
Magnetic resonance imaging performed on day of life 8 demonstrating lissencephaly, a classical finding in patients with Miller-Dieker syndrome. Anteroposterior view on the left and lateral view on the right.

On the day of the procedure, the patient’s mother endorsed a recent diagnosis of pneumonia for which he was currently on a course of oral antibiotic treatment. Outpatient medications at that time included as needed intranasal midazolam for seizures, scopolamine, valproic acid, levetiracetam, topiramate, gabapentin, and an as needed albuterol nebulizer. His past surgical history included circumcision, laparoscopic gastrostomy tube placement, adenoidectomy, tonsillectomy, cystoscopy with ureteroscopy, lithotripsy, and stone retraction. There was no history of previous perioperative anesthetic concerns although previous attempts at airway instrumentation with direct laryngoscopy revealed a grade IIb or IIa view of the glottis. The family history was negative for anesthetic complications.

The patient’s preoperative vital signs were all normal: temperature 36.2 °C, pulse 94 beats/min, respirations 22 breaths/min, blood pressure 95/59 mm Hg, and SpO_2_ 100% on room air. Documented preoperative physical exam findings related to MDS included micro/retrognathia and a high arched palate. Cardiac and respiratory examinations were unremarkable. He was assigned an American Society of Anesthesiologists (ASA) physical status 3 classification, and special concerns included possible difficult airway management, difficult vascular access, potential for postoperative hospital admission, and possible need for postoperative endotracheal intubation and mechanical ventilation. The anesthetic plan included general anesthesia with endotracheal intubation with the above noted concerns. The patient was held *nil per os* (NPO) for 6 h other than his routine morning medications including his anticonvulsant medications. He was transported to the operating room where routine ASA monitors were placed. Anesthesia was induced by the inhalation of incremental concentrations of sevoflurane in 100% oxygen. Following the induction of anesthesia, a peripheral intravenous (IV) cannula was placed. Following the verification of adequate bag-valve-mask ventilation, fentanyl (2 µg/kg) and rocuronium (0.6 mg/kg) were administered. Indirect video laryngoscopy with a CMAC (Macintosh 2 blade) provided a grade I view of his glottis, and his trachea was intubated on the first attempt with a 4.5-mm cuffed endotracheal tube (ETT), which was then taped at a depth of 14.5 cm. The cuff was inflated to seal the airway at 20 cm H_2_O. Maintenance of general anesthesia was provided by sevoflurane (expired concentration 1.5–2%) in air/oxygen. Ciprofloxacin was administered for perioperative antibiotic management. The surgical procedure lasted approximately 75 min. There was minimal blood loss, and intraoperative fluids included 400 mL of lactated Ringer’s. Additional intraoperative medications included dexamethasone (0.15 mg/kg), ondansetron (0.15 mg/kg), and ketorolac (0.5 mg/kg). At the completion of the surgical procedure, residual neuromuscular blockade was reversed with sugammadex (4 mg/kg). During emergence, bronchospasm was treated with a single dose of IV epinephrine (1 µg/kg). After the patient had emerged from anesthesia, his trachea was extubated. The patient was transported to the post-anesthesia care unit and then discharged to phase II recovery. His postoperative course was unremarkable, and he was discharged home later that day. The post-procedure anesthetic evaluation revealed acceptable respiratory, cardiovascular, and neurological assessment in addition to appropriate pain and nausea control.

## Discussion

Given its rarity in the general population, there remains limited and primarily anecdotal information regarding the perioperative care of children with MDS. Our review of the literature identified only two previous reports of anesthetic care in these patients [8, 9]. The first case, published in 2015, outlined anesthetic care for an 18-month-old female patient with MDS who underwent a general anesthetic for elective laryngotracheal separation and percutaneous gastrotomy [8]. On preoperative examination, the patient was noted to have facial features consistent with MDS, similar to our patient, and for this reason preparation was taken in anticipation of a difficult airway. Two attending anesthesiologists were present for anesthetic induction, airway management, and endotracheal intubation. Specialized airway equipment for difficult airway management was readily available. Of note, a bispectral index (BIS) monitor was placed prior to the induction of anesthesia, and a low unanesthetized reading was noted (BIS value of 16–21) prior to the administration of any anesthetic medications. Anesthesia was induced by the IV administration of midazolam (0.3 mg/kg) and fentanyl (3 µg/kg). Bag-valve-mask ventilation was not problematic, and after the administration of rocuronium (1 mg/kg), the trachea was intubated with a 4.0-mm uncuffed ETT using direct laryngoscopy with a Macintosh 2 laryngoscope blade. The vocal cords were easily visualized. An air leak was noted at 20 cm H_2_O. Maintenance anesthesia included sevoflurane (2.5%) and remifentanil (0.1–0.2 µg/kg/min). Intraoperative BIS values varied from 15 to 34 while postoperative BIS levels remained low ranging from 26 to 33.

A second case report, published in 2023, outlined the anesthetic management of a 4-year-old girl (14 kg) with MDS presenting for exotropia surgery [9]. This patient had similar facial abnormalities as those described in our case and the previous case report discussed above. Additional comorbid conditions included a patent foramen ovale and a history of tonic-clonic seizures. She had been seizure free for 1 year prior to undergoing anesthesia. Unlike the report of Wakiguchi et al [8], the patient’s trachea was intubated using an indirect video laryngoscopy (Glidescope) following the administration of thiopental (75 mg) and rocuronium (10 mg) with a Cormack Lehane grade 2 view. The epiglottis was noted to be large and curvilinear. As was previously reported, the awake BIS value was low at 37. BIS values throughout the case ranged from 17 to 26 during maintenance anesthesia that included sevoflurane and remifentanil. The BIS values returned to 51 after she had recovered from the anesthetic. No other perioperative challenges or adverse effects were noted.

These two case reports highlight the potential perioperative challenges of children with MDS including challenging airway management, associated congenital heart disease, and CNS disabilities including seizure disorders, poor upper airway control, aspiration risk, and hypotonia. Similar concerns and challenges were identified in our patient, which adds to the published experience of the perioperative care of these patients. As with all anesthetic care, the first step involves a focused history and physical examination with identification of end-organ involvement and potential challenges for anesthetic care. Patients with known genetic syndromes pose a variety of challenges to the anesthesia provider, including difficulties with airway management, bag-valve-mask ventilation or endotracheal intubation [10, 11]. This is a concern in patients with MDS, as facial dysmorphism is a hallmark phenotypic presentation with distinctive craniofacial and midface features that may complicate bag-valve-mask ventilation, direct laryngoscopy, and endotracheal intubation. The preoperative airway evaluation in our patient was limited by cognitive impairment, but physical examination revealed micrognathia and a small mouth opening. During previous anesthetic care in our patient, the potential for difficulties with direct laryngoscopy and endotracheal intubation had been known, as airway instrumentation revealed a grade IIa or IIb view of the glottis. Given these concerns in our patient, spontaneous ventilation was maintained, and neuromuscular blockade was administered only after the adequacy of bag-valve-mask ventilation was verified. Additionally, the equipment required for dealing with the difficult airway, including indirect video laryngoscopy and the difficult airway cart, was available in the room during anesthetic induction [12, 13]. The trachea was intubated uneventfully with a grade I view of the glottis using indirect video laryngoscopy.

An additional concern in patients with MDS is the presence of associated congenital heart defects. These most common defects include conotruncal defects (tetralogy of Fallot or double outlet right ventricle), atrial or ventricular septal defects, patent ductus arteriosus, or rare coronary artery involvement such as anomalous right coronary artery from the pulmonary artery. Given these concerns, routine screening with echocardiography and an electrocardiogram is recommended prior to anesthetic care. Depending on the results of pre-anesthesia testing, invasive hemodynamic monitoring may be indicated for specific surgical procedures.

Structural involvement of the CNS, such as lissencephaly, is a consistent finding in MDS patients. Associated comorbid conditions include seizure disorder, developmental delay with cognitive impairment, and hypotonia. Seizure disorders are a frequent comorbid condition, which may impact perioperative care. Patients should continue their routine antiepileptic drugs (AEDs) during the perioperative period, including on the morning of surgery, regardless of NPO status [14]. Both the inhalational and IV anesthetic agents (e.g., propofol) have anticonvulsant properties, and there is limited need to adjust the choice of anesthetic agents for premedication, induction, and maintenance of anesthesia in patients with an underlying seizure disorder [15]. As the physiologic stress of surgery may lower the seizure threshold, postoperative monitoring may be indicated to identify break-through seizures. Routine AEDs should be continued during the perioperative period, with IV alternatives substituted if oral administration is not feasible. Chronic AED administration may affect the metabolism and dosing of various anesthetic agents, opioids, and neuromuscular blocking agents (NMBAs) [16].

As described in both referenced case reports above, and in other cases of lissencephaly-associated syndromes, the BIS values prior to, during, and in response to anesthetic agents will be lower than what would be expected in the general population [8, 9]. Valkenburg et al who described similar findings in a patient with lissencephaly and West syndrome theorized that electroencephalographic abnormalities secondary to associated neurologic disorders, structural anomalies of the brain, and anticonvulsant use may contribute to the abnormally low values [17]. Although not a routine feature of intraoperative care, depth of anesthesia monitoring has increased in pediatric-aged patients, especially those ≥ 6 years of age [18]. However, as noted above, BIS monitoring is likely to be unreliable in patients with MDS.

Patients with MDS often present with associated hypotonia related to the underlying genetic defect and associated CNS involvement. The associated hypotonia and poor upper airway control increases the risk of perioperative upper airway obstruction or aspiration, which may result in postoperative respiratory insufficiency. These concerns may be further exacerbated by the impact of the residual effects of anesthetic agents on respiratory function and airway control. Short-acting anesthetic agents (desflurane/sevoflurane and remifentanil) are recommended to avoid the impact of residual anesthetic effects on postoperative respiratory function. Adhering to the appropriate fasting guidelines prior to anesthetic care is mandatory. Rapid sequence intubation may be indicated in patients with clinically significant reflux with the consideration of risk-benefit ratio of this practice in patients in whom endotracheal intubation may be problematic. Given the limited clinical concerns of aspiration and the potential for difficulties with airway management in our patient, inhalation induction with the maintenance of spontaneous ventilation was chosen. In patients with significant upper airway obstruction or following prolonged procedures, postoperative monitoring of respiratory function in an intensive care unit (ICU) setting may be indicated.

Given the paucity of case reports regarding anesthetic care in patients with MDS, there is limited evidence-based medicine to guide the choice of NMBA. Depolarizing NMBA, such as succinylcholine, should theoretically be avoided in patients with hypotonia due to concerns for rhabdomyolysis and hyperkalemia [19]. The response to and duration of non-depolarizing NMBAs may be exaggerated [20, 21]. In our patient, a single intraoperative dose of rocuronium (0.6 mg/kg) was administered to facilitate endotracheal intubation. In other clinical scenarios for more prolonged procedures when repeated doses of NMBAs are administered, train-of-four monitoring may be indicated to guide dosing of NMBAs and documentation of full reversal. In the setting of hypotonia, reversal of residual neuromuscular blockade with sugammadex may ensure full reversal [22].

Hypotonia may lead to contractures, difficult vascular access or concerns during positioning. Ultrasound guidance may be necessary in MDS patients to facilitate peripheral venous or arterial access [23]. Padding of bony prominences is necessary to prevent skin breakdown during prolonged procedures or immobilization. Growth failure, nutritional issues, and CNS disabilities may predispose to hypothermia. Maintenance of normothermia may require the use of overhead heating lights, forced air warming devices, and higher room temperature. Sacral dimpling is also a relatively common manifestation in patients with MDS [24]. Sacral dimples may be associated with an underlying dermal sinus tract or abnormal spinal cord/vertebral anatomy that may complicate or contraindicate neuraxial anesthesia [25]. Point-of-care ultrasound may be considered to guide needle placement in this scenario [26].

### Learning points

In summary, we present the successful anesthetic management of a child with MDS. Children with this congenital disorder suffer from lissencephaly, facial dysmorphisms, neurologic impairments, and associated congenital heart disease, which may require special consideration during perioperative care. As previous reports of anesthetic care are limited to only two previous anecdotal reports, there is limited evidence-based medication to guide perioperative care. Of primary importance is the potential for difficulties with airway management related to facial dysmorphism. CNS disabilities may include poor upper airway control, hypotonia, seizures, and cognitive impairment. Additional comorbid conditions include associated congenital heart disease, generally conotruncal or septal involvement. Effective and safe perioperative care requires identification of these concerns during the preoperative examination with preparation to limit impact of these conditions on perioperative care.

## Data Availability

Any inquiries regarding supporting data availability of this study should be directed to the corresponding author.
